# Insufficient activation of autophagy allows accumulation of cellular damage and may contribute to sustained organ failure in prolonged critically ill patients

**DOI:** 10.1186/cc9806

**Published:** 2011-03-11

**Authors:** J Gunst, S Derde, I Derese, M Boussemaere, F Güiza, W Martinet, JP Timmermans, A D'Hoore, PJ Wouters, G Van den Berghe, I Vanhorebeek

**Affiliations:** 1 KULeuven, Leuven, Belgium; 2 Universiteit Antwerpen, Antwerp, Belgium

## Introduction

Prolonged critically ill patients face a high risk of death, which is most often due to nonresolving multiple organ failure and muscle weakness. Increased oxidative stress, accumulation of damaged proteins and mitochondrial dysfunction contribute to cellular and organ dysfunction, and persistence of these abnormalities may trigger additional damage. Autophagy is the only degradation pathway able to remove toxic protein aggregates and damaged mitochondria. Feeding and insulin are two powerful suppressors of autophagy. We therefore hypothesized that in fed, prolonged critically ill patients receiving insulin, the required activation of autophagy to clear cellular damage could be impaired.

## Methods

We studied autophagy in liver and skeletal muscle biopsies from fed, prolonged critically ill patients, in whom hyperglycemia was tolerated or treated with insulin in the context of two randomized, clinical studies on intensive insulin therapy [1,2], as compared with biopsies from matched controls. We quantified (ultra)structural abnormalities and hepatic and skeletal muscle protein levels of key players in autophagy.

## Results

Morphologically, both liver and muscle revealed an autophagy-deficiency phenotype. Proteins involved in initiation and elongation steps of autophagy were induced 1.3-fold to 6.5-fold by critical illness (*P *≤ 0.01), but mature autophagic vacuole formation was 62% impaired (*P *= 0.05) and proteins normally degraded by autophagy accumulated up to 97-fold (*P *≤ 0.03). Markers of mitophagy (selective autophagy of mitochondria) were unaltered or downregulated (*P *= 0.05). Although insulin preserved hepatocytic mitochondrial integrity (*P *= 0.05), it further reduced the number of autophagic vacuoles by 80% (*P *= 0.05).

## Conclusions

Activation of autophagy appeared insufficient in liver and skeletal muscle biopsies from prolonged critically ill patients and may be further suppressed by insulin treatment. Incomplete clearance of cellular damage inflicted by illness and aggravated by hyperglycemia could explain lack of recovery from organ failure in critically ill patients. These data open perspectives for therapies that activate autophagy during critical illness.
